# Structural Evolution of α-Fe_2_O_3_(0001) Surfaces Under Reduction Conditions Monitored by Infrared Spectroscopy

**DOI:** 10.3389/fchem.2019.00451

**Published:** 2019-06-25

**Authors:** Ludger Schöttner, Alexei Nefedov, Chengwu Yang, Stefan Heissler, Yuemin Wang, Christof Wöll

**Affiliations:** Institute of Functional Interfaces (IFG), Karlsruhe Institute of Technology, Karlsruhe, Germany

**Keywords:** iron oxide, CO adsorbed infrared spectroscopy, IR spectroscopy, reduced oxides, XPS

## Abstract

The precise determination of the surface structure of iron oxides (hematite and magnetite) is a vital prerequisite to understand their unique chemical and physical properties under different conditions. Here, the atomic structure evolution of the hematite (0001) surface under reducing conditions was tracked by polarization-resolved infrared reflection absorption spectroscopy (IRRAS) using carbon monoxide (CO) as a probe molecule. The frequency and intensity of the CO stretch vibration is extremely sensitive to the valence state and electronic environments of surface iron cations. Our comprehensive IRRAS results provide direct evidence that the monocrystalline, stoichiometric α-Fe_2_O_3_(0001) surface is single Fe-terminated. The initial reduction induced by annealing at elevated temperatures produces surface oxygen vacancies, where the excess electrons are localized at adjacent subsurface iron ions (5-fold coordinated). A massive surface restructuring occurs upon further reduction by exposing to atomic hydrogen followed by Ar^+^-sputtering and annealing under oxygen poor conditions. The restructured surface is identified as a Fe_3_O_4_(111)/Fe_1−x_O(111)-biphase exposing both, Fe^3+^ and Fe^2+^ surface species. Here the well-defined surface domains of Fe_3_O_4_(111) exhibit a Fe_oct2_-termination, while the reduced Fe_1−x_O(111) is Fe^2+^(oct)-terminated. These findings are supported by reference IRRAS data acquired for CO adsorption on magnetite (111) and (001) monocrystalline surfaces.

## Introduction

Although iron oxides have been the topic of numerous studies, their surface is of complex nature and their structure depends strongly on the interaction with the ambience (Kuhlenbeck et al., 2013; Parkinson, 2016). Not only because of the comprehensive phase diagram and the ability to form polymorphs, but also because of the unique material properties including magnetism, the surface properties of iron oxide are among the most challenging research fields in inorganic chemistry and solid-state physics. In previous works, a number of structural conversions and surface reconstructions have been reported (Ketteler et al., 2001; Weiss and Ranke, 2002; Sakurai et al., 2009; Kuhlenbeck et al., 2013; Parkinson, 2016). It is therefore not surprising that the discussion on many common iron oxide facets toward surface stabilization and termination has remained controversial. For detailed insights, we refer the reader to two recent excellent review papers (Kuhlenbeck et al., 2013; Parkinson, 2016). In section Preview on Related Iron Oxide Crystal Structures, we have briefly summarized the important experimental and theoretical results reported in the literature for three relevant iron oxide surfaces [α-Fe_2_O_3_(0001), Fe_3_O_4_(111), Fe_3_O_4_(001)] in the form of both thin films and single crystals.

The chemical and physical properties of metal oxides strongly depend on the structural arrangement of atoms at their surfaces (Wang and Wöll, 2017). Furthermore, it has been shown that the chemical reactivity can be dramatically changed in the presence of defects like oxygen vacancies. Especially under reducing conditions there are numerous such defects, with their densities depending on temperature and the surrounding atmosphere (Parkinson, 2016). However, it is difficult to gain atomic-level information on metal oxide surface structures since some of the standard methods in surface science cannot be applied in a straightforward fashion, e.g., due to charging problems.

This problem can be avoided when using non-destructive surface analysis methods insensitive to charging effects. An important example is infrared (IR) spectroscopy. In combination with appropriate probe molecules like carbon monoxide (CO), this vibrational spectroscopy has been shown to provide insights into structural, electronic and reactive properties of model and nanostructured catalysts (Yang and Wöll, 2017). In addition, the interaction between CO and iron oxide (α-Fe_2_O_3_, hematite, Fe_3_O_4_, magnetite, Fe_1−x_O, wüstite) is of great interest in itself because of detailed knowledge about the binding of CO to iron oxides is required for a fundamental understanding of industrial catalytic processes such as CO oxidation and water gas-shift reactions (de Smit and Weckhuysen, 2008; Freund et al., 2011; Zhu and Wachs, 2016; Wei et al., 2018). The adsorption of CO on various well-defined metal surfaces has been extensively studied by infrared reflection absorption spectroscopy (IRRAS). However, the application of this approach on metal oxide single-crystal surfaces is challenging due to the inherent experimental difficulties arising from the low reflectivity of dielectric substrates in the IR regime. Recent progress in instrumentation, however, has allowed us to obtain high-resolution data for a number of different oxide surfaces (e.g., CeO_2_, TiO_2_, ZnO, α-Fe_2_O_3_) (Buchholz et al., 2016; Yu et al., 2016; Yang et al., 2017a,b; Chen et al., 2019; Schöttner et al., 2019). These IRRAS results have demonstrated that CO is well suited as a probe molecule to monitor the atomic structure evolution of oxide surfaces under different conditions. In particular, defects like oxygen vacancies and surface restructuring could be clearly identified (Xu et al., 2012; Yang et al., 2014, 2017a,b).

To the best of our knowledge, no IRRAS data of CO adsorption on surfaces of macroscopic iron oxide single crystals have been reported so far. Several studies have been reported on related systems including CO adsorbed on α-Fe_2_O_3_ powders (Zecchina et al., 1988) and Fe_3_O_4_(111) thin films grown on Pt(111) (Lemire et al., 2004, 2005; Kuhlenbeck et al., 2013; Li et al., 2018). Here, we present a thorough polarization-resolved IRRAS study of CO adsorption on differently treated iron (III/II) oxide single-crystal surfaces, focusing on α-Fe_2_O_3_(0001) that has shown to be the most abundant facet exposed by hematite nanoparticles (Hartman, 1989). Our systematic IRRAS results allow to gain detailed insight into the structural evolution of the α-Fe_2_O_3_(0001) surface under reducing conditions. The assignment of the IR bands was assisted by additional reference measurements of CO adsorption on well-defined Fe_3_O_4_(111) and Fe_3_O_4_(001) monocrystalline substrates.

## Models And Methods

### Preview on Related Iron Oxide Crystal Structures

**Figure 1:**
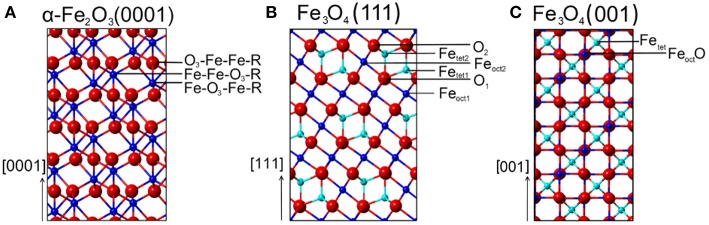
Schematic representations (side view) of various surface terminations for **(A)** α-Fe_2_O_3_(0001), **(B)** Fe_3_O_4_(111), and **(C)** Fe_3_O_4_(001) surfaces. Red spheres correspond to oxide lattice ions; dark blue spheres and cyan spheres represent iron ions on octahedral (oct) and tetrahedral (tet) lattice sites, respectively.

α-Fe_2_O_3_ crystallizes in a corundum structure characterized by a slightly distorted hexagonal close-packed oxide-ion lattice where 2/3 of the octahedral sites are occupied by Fe^3+^ ions with non-degenerated energy levels on metal d-electrons due to a ligand field splitting originating from Fe-O hybridization (Figure S1) (de Groot et al., 1989). Oxygen ions that are located parallel to (0001) plane levels, are separated by an iron double layer, yielding a stacking sequence of O_3_-Fe-Fe repeat units. There are three different surface terminations possible for α-Fe_2_O_3_(0001): a single iron layer (Fe-O_3_-Fe-R), a double iron layer (Fe-Fe-O_3_-R) and an oxygen (O_3_-Fe-Fe-R) layer (Figure 1A).

It is known that the structural and electronic properties of hematite (0001) depends strongly on the interaction with the ambience and the preparation parameters (Ketteler et al., 2001; Weiss and Ranke, 2002; Kuhlenbeck et al., 2013; Parkinson, 2016). In particular, the surface termination α-Fe_2_O_3_(0001) is highly sensitive to the oxygen partial pressure and preparation temperature. Freund and coworkers have systematically studied the surface structures of α-Fe_2_O_3_(0001) thin films supported on Pt(111) by scanning tunneling microscopy (STM) and IRRAS (Lemire et al., 2005). The results revealed a ferryl (Fe = O) termination coexisting with domains of the Fe-terminated surface in 10^−3^-1 mbar O_2_ at ~1,050 K, while the preparation at higher pressures of O_2_ (>1 mbar) yields O-terminated surfaces (Shaikhutdinov and Weiss, 1999). In contrast, an exclusive Fe-termination was reported on thin films (Thevuthasan et al., 1999) as well as on single crystal substrates under O-deficient conditions (<10^−5^ mbar) (Yamamoto, 2010; Schöttner et al., 2019). For α-Fe_2_O_3_(0001) single crystals, it has been reported that Ar^+^-sputtering followed by annealing in 10^−6^ mbar of O_2_ at about 1,000 K leads to a massive surface restructuring, forming a Fe_3_O_4_(111) layer (Condon et al., 1994, 1998; Camillone et al., 2002). A biphase structure was observed upon heating in oxygen (10^−6^ mbar) to higher temperatures (~1,100 K) (Condon et al., 1995, 1998; Camillone et al., 2002). The STM results revealed the formation of long-range order of α-Fe_2_O_3_(0001) and Fe_1−x_O(111) domains, which are characterized by floret-like spots in the low-energy electron diffraction (LEED) pattern described as (√3 × √3)R30° reconstruction (Kurtz and Henrich, 1983; Lad and Henrich, 1988; Condon et al., 1995). In addition, the surface chemistry and physics of α-Fe_2_O_3_(0001) has been studied by a number of theoretical groups [for details see the review by Parkinson, 2016] (Wang et al., 1998; Bergermayer et al., 2004; Trainor et al., 2004; Nguyen et al., 2014; Lewandowski et al., 2016; Ovcharenko et al., 2016).

In the crystal structure of Fe_3_O_4_ oxygen anions are aligned in a cubic closed packing of equal spheres, where Fe^3+^ cations are located at tetrahedrally coordinated interstitial sites. Other cations are located at octahedrally coordinated lattice positions, among which 50% exhibit the valence state +3 while the other 50% are in the +2 oxidation state. This chemical composition is typical for inverse spinel bulk structures and can be written as Fe^3+^[Fe^3+^Fe^2+^]O_4_ to illustrate that the first type is tetrahedrally coordinated (A-type), whereas the parenthesized Fe^2+^ and Fe^3+^ ions occupy octahedrally coordinated sites (B-type). At low-pressure and low-temperature (CO measurement conditions), Fe_3_O_4_ consists of a crystal structure with monoclinic symmetry (Wright et al., 2002; Bengtson et al., 2013). Above 120 K, known as the Verwey temperature (Verwey, 1939), the octahedrally coordinated iron atoms in magnetite have an average oxidation state of Fe +2.5 due to electron hopping between equal numbers of Fe^2+^ and Fe^3+^ ions. Such electron movement is frozen in Fe_3_O_4_ below 120 K and therefore accompanied by a significant decrease in the electrical conductivity.

For Fe_3_O_4_(111) surfaces, six terminations may occur when cleaving the (111) stacking sequence (Figure 1B). The different terminations exhibit Fe atoms on octahedral (Fe_oct1_ or Fe_oct2_) and tetrahedral (Fe_tet1_ or Fe_tet2_) lattice sites, as well as O layers (O_1_ or O_2_). The surface structure of Fe_3_O_4_(111) has been the subject of numerous experimental and theoretical investigations. Quantum chemical calculations have predicted that the Fe_oct2_-termination is energetically favored on Fe_3_O_4_(111) under oxygen-deficient conditions, followed closely by the Fe_tet1_ termination (Ahdjoudj et al., 1999; Zhu et al., 2006; Grillo et al., 2008; Noh et al., 2015). Recent work combining IRRAS and DFT (Li et al., 2018) revealed that the regular Fe_3_O_4_(111) thin film is Fe_tet1_-terminated over close-packed oxygen layer, in line with the LEED *IV* results (Ritter and Weiss, 1999; Sala et al., 2012). STM studies provided further evidence for the Fe_tet1_ termination on the pristine Fe_3_O_4_(111) surface, while the Fe_oct2_ termination was observed under oxygen-poor conditions (Lennie et al., 1996; Shimizu et al., 2010). In addition, a long-range ordered biphase structure was reported for reduced Fe_3_O_4_(111) in the form of both thin films (Shaikhutdinov et al., 1999) and single-crystals (Condon et al., 1997; Paul et al., 2007). The STM and LEED data suggested the coexistence of Fe_3_O_4_(111) and Fe_1−x_O(111) domains.

From the ideal Fe_3_O_4_(001) model, only two surface terminations are possible, namely Fe_tet_ and Fe_oct_O layers (Figure 1C). Again, the surface structure of Fe_3_O_4_(001) depends strongly on the preparation parameters (Tarrach et al., 1993; Parkinson et al., 2011; Bartelt et al., 2013). The polar Fe_3_O_4_(001) surface is well-known to be stabilized by a (√2 × √2)R45° reconstruction. The reconstructed surface was first studied using STM by Wiesendanger et al. (1992) and later by other groups [see e.g., Chambers and Joyce, 1999; Rustad et al., 1999; Stanka et al., 2000; Mariotto et al., 2002]. Recently, Parkinson et al. (Bliem et al., 2014) proposed a subsurface cation vacancy (SCV) model by using STM in conjunction with LEED *IV* analysis and theory. In this model, the reconstructed surface features a Fe_oct_O termination (B-layer) and is slightly distorted by a rearrangement of the cations in the subsurface layers.

Overall, due to the great complexity, an unambiguous experimental determination of the surface atomic structure for these iron oxides under different conditions is still a challenging task.

### Experimental Details

The IR measurements were carried out in an UHV multi-chamber system (Prevac). The base pressure of the entire chambers is in 10^−10^ mbar range. The IR spectra were recorded in reflection absorption mode using a state-of-the-art Fourier-Transform (FT)-IR spectrometer (Bruker Vertex 80v) that is directly coupled to the UHV chamber via differentially pumped KBr-windows. The IR-spectrometer contains an internal polarizer module, which allows to carry out polarization-resolved IRRAS experiments. All CO adsorption measurements reported here were conducted for the respective iron oxide substrate mounted in a cryo-pumped IR-chamber at low temperatures. With liquid-He cooling, we could achieve sample temperatures of 65 K, which was sufficiently low to yield CO adsorption on all substrates studied here. Exposures to CO were carried out by using a leak-valve based directional doser. Gas dosages are quoted in Langmuir (1 L = 1.33·10^−6^ mbar·s). The IR spectra were recorded after saturating the iron oxide surfaces with CO (1 L). The polarization-dependent IRRAS measurements were conducted with p- and/or s-polarized light at grazing incidence, with the angle of 80° with respect to the surface normal. The IRRA spectra are shown in absorbance mode. The resolution of the IR spectrometer was set to 4 cm^−1^ and 2,048 scans were accumulated for a single spectrum.

Iron oxide single crystals were mounted on a sample holder dedicated for e-beam heating. Temperatures were measured with a NiAl-NiCr thermocouple, that is spot-welded at a position between heating plate and backside of the sample. Surface quality and cleanliness were checked by XPS and LEED. Supplementary XPS measurements on the oxidation states of iron atoms for differently prepared iron oxide surfaces were conducted at the HESGM-beamline of the BESSY II storage ring, which is a part of the Helmholtz-Center Berlin.

The iron oxide single crystals used in this study were cut from minerals and polished (SurfaceNet GmbH). The α-Fe_2_O_3_(0001) substrate was cleaned by repeated cycles of annealing with stepwise heating from 850 K to 950 K in 10^−5^ mbar O_2_ (Supporting information, Figures S2A–C) (Lübbe and Moritz, 2009; Schöttner et al., 2019). The slightly reduced Fe_2_O_3_(0001) was formed after over-annealing the sample by gradual heating from 950 K to 1,050 K in 10^−5^ mbar O_2_. A highly reduced surface was prepared by exposing the clean and stoichiometric α-Fe_2_O_3_(0001) to atomic hydrogen followed by post-annealing at 1,000 K. Then the reduced α-Fe_2_O_3_(0001) surface was prepared by repeated cycles of sputtering with 1.0 keV Ar^+^ for 15 min and subsequent annealing at 950 K in 10^−6^ mbar oxygen for 20 min until a LEED pattern gets visible.

The Fe_3_O_4_(111) single crystal surface was prepared by sputtering with 1.0 keV Ar^+^ for 15 min and subsequent annealing at 920 K in 10^−6^ mbar oxygen for 20 min. The surface of Fe_3_O_4_(001) was cleaned by Ar^+^-sputtering at 1.0 keV for 15 min and annealing at 870 K in a background of oxygen at 10^−6^ mbar for 20 min (Figure S3) (Nie et al., 2013).

## Results And Discussion

### CO Adsorption on the Pristine α-Fe_2_O_3_(0001) Surface

**Figure 2:**
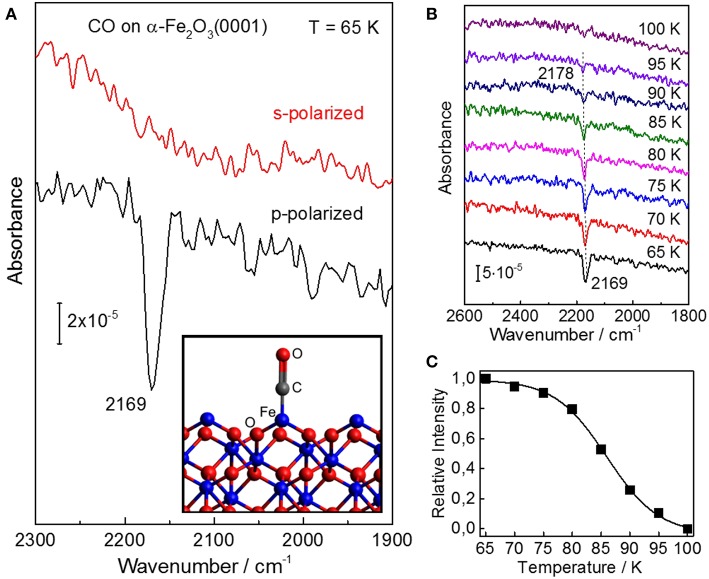
IRRAS results for CO adsorption on the well-ordered stoichiometric α-Fe_2_O_3_(0001) surface. **(A)** Polarization-dependent IRRAS data recorded after exposing the clean surface of α-Fe_2_O_3_(0001) to 1 L CO at 65 K. The adsorption model is shown as inset. **(B)** Temperature-dependent IRRAS data starting from a saturated CO coverage at 65 K. Here, all spectra were recorded at an incidence angle of 80° with p-polarized light. **(C)** The relative CO band-intensity is shown as a function of the sample temperature.

In Figure 2 we display polarization-resolved IRRAS data recorded after a saturating exposure of the pristine stoichiometric α-Fe_2_O_3_(0001) single crystal surface to CO at 65 K. For p-polarized light, we observe a single, symmetric band at 2,169 cm^−1^ which is characteristic for CO bound to the coordinatively unsaturated surface Fe^3+^ cations (Zecchina et al., 1988), indicating the presence of a well-defined surface with only one adsorption site. As mentioned above, the surface termination of iron oxides depends strongly on the preparation conditions (Kuhlenbeck et al., 2013; Parkinson, 2016). For example, the ferryl termination was observed for α-Fe_2_O_3_(0001) thin films after oxidation with O_2_ partial pressures of up to 1 mbar, showing a typical IR band at 2,185 cm^−1^ (Lemire et al., 2005), which is not seen in the present IR spectra (Figure 2). Our IRRAS data provides direct spectroscopic evidence that the pristine α-Fe_2_O_3_(0001) surface is single Fe^3+^-terminated.

The difference between p- and s-polarizations can be understood by considering the transition dipole moment (TDM) describing the vibrational excitation of CO. The intensity and sign of an IR peak is given by the interaction of the TDM with the vector of the E-field. Note that for metal surfaces including metal-supported oxide thin films, IRRAS data can only be obtained for IR light polarized perpendicular to the surface according to the so-called surface selection rule. In the case of dielectric bulk materials (e.g., metal oxide single crystals), however, both s- and p-polarized components can interact with adsorbate vibrations being highly dependent on the orientation of TDM, thus allowing for a determination of the adsorption geometry (Wang and Wöll, 2017). The absorbance bands excited by E_*s*_ are always negative, while the vibrations excited by p-polarized components (normal E_*p, n*_ and tangential E_*p, t*_, showing always opposite signs) can be negative or positive depending on the incidence angle Θ and the refractive index *n* of the substrate (Brunner et al., 1997).

For the adsorption of a full CO monolayer on α-Fe_2_O_3_(0001) at 65 K, a single IR band with negative sign was observed at 2,169 cm^−1^ for p-polarized light but not for s-polarized light (Figure 2A). Since the TDM is oriented parallel to the CO molecular axis, these polarization-dependent IRRAS results indicate that CO is bound to surface Fe^3+^ ions in an upright geometry. Furthermore, we observed a coverage-dependent frequency shift (Figure S4), in line with IR results reported for CO adsorbed on α-Fe_2_O_3_ powder samples (Zecchina et al., 1988). This coverage-induced frequency shift can be explained in terms of lateral adsorbate-adsorbate interactions including both dynamic and substrate-mediated static effects. For CO adsorption on oxide surfaces, the substrate mediated interactions (static shift) play a predominant role, whereas the contribution from dynamic interactions originating from the dipole-dipole coupling between CO adsorbates is rather small (Mahan and Lucas, 1978; Hollins and Pritchard, 1980).

The coverage-dependent frequency shift in CO stretch frequency was also observed after heating the sample to higher temperatures, i.e., with decreasing the CO coverage (temperature-induced CO desorption). The intensity of the CO vibrational band remains stable on α-Fe_2_O_3_(0001) up to 70 K (Figure 2B) but strongly decreases upon further heating. The IR band has disappeared at 100 K, indicating complete desorption of CO. The thermal desorption of CO is accompanied by a blue-shift in frequency from 2,169 to 2,178 cm^−1^. A quantitative analysis of the temperature-dependent IRRAS results (CO coverage as a function of temperature, see Figure 2C) (Gottfried et al., 2003) yields an activation energy for CO desorption on α-Fe_2_O_3_(0001) of 24 kJ·mol^−1^ (using a frequency factor of 10^13^ s^−1^), demonstrating that CO binds only weakly to the surface Fe^3+^ ions.

### CO Adsorption on Reduced α-Fe_2_O_3_(0001)

**Figure 3:**
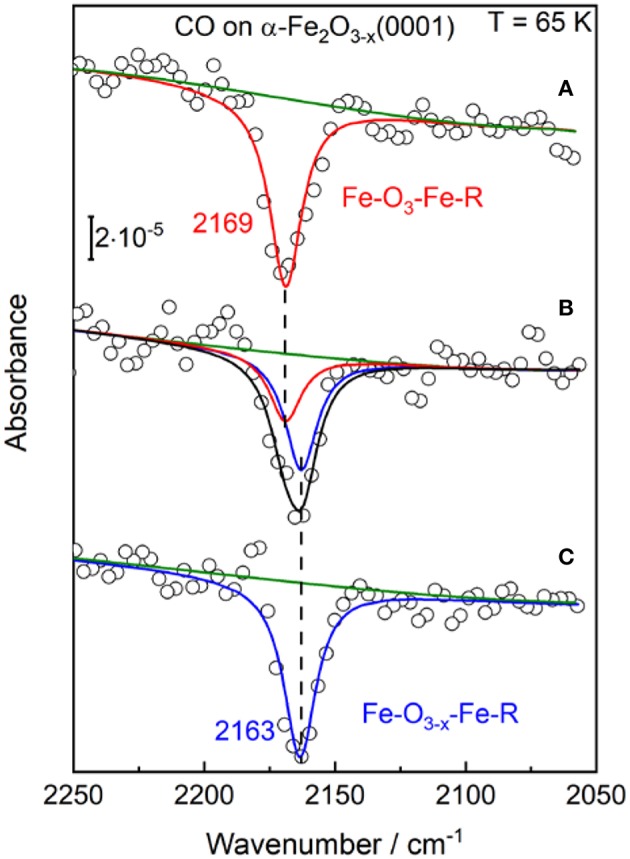
The p-polarized IRRA spectra obtained after saturation adsorption of CO (1 L) on α-Fe_2_O_3_(0001) at 65 K. The α-Fe_2_O_3_(0001) samples were pretreated in 10^−5^ mbar O_2_ at different temperatures: **(A)** 850–950 K; **(B)** 950–1,000 K; **(C)** 1,000–1,050 K.

In the next step the stoichiometric α-Fe_2_O_3_(0001) was subjected to annealing in 10^−5^ mbar oxygen at higher temperatures increased stepwise from 850 K to 1050 K. The corresponding structure evolution was tracked by measuring the vibrational frequency of adsorbed CO. Upon annealing to 950 K, the IRRA spectrum shows only a single CO band at 2,169 cm^−1^ (Figure 3), assigned to CO adsorbed at the 3-fold coordinated (Fe-O_3_) Fe^3+^ sites of the stoichiometric α-Fe_2_O_3_(0001) surface, as discussed above. A new CO band at 2,163 cm^−1^ appears and becomes the dominating one when increasing the sample temperature to 950–1000 K under O-poor conditions. After heating the sample to 1,000–1,050 K, only the 2,163 cm^−1^ band is detected, while the Fe^3+^-related CO vibration at 2,169 cm^−1^ observed for the fully oxidized surface disappears completely (see Figure 3). Note that in the fitting process, the half width for all CO peaks was set to be equal.

Overall, the present IRRAS data indicate the presence of surface oxygen vacancies (V_O_) created by annealing to elevated temperatures. Formation of V_O_ defects have been found to induce a red-shift of CO frequencies on other oxides [e.g., rutile TiO_2_(110) surface; Xu et al., 2012]. Here, the slightly reduced α-Fe_2_O_3_(0001) surface (Fe-O_3−x_-Fe-R) is characterized by a CO band position of 2,163 cm^−1^, which we assign to CO bound to 2-fold coordinated (Fe-O_2_) Fe^3+^ ions adjacent to O-vacancy sites. Importantly, the rather small red shift of 6 cm^−1^ stands for an only little electronic structure modification of surface iron cations, suggesting that the excess electrons are not trapped at the 2-fold coordinated surface Fe^3+^ ions. This finding is in excellent agreement with the DFT calculations made on the reduced α-Fe_2_O_3_(0001) surface (Ovcharenko et al., 2016). Along with the removal of a surface oxygen atom, two excess electrons were predicted to occupy the easily available Fe 3d states on two neighboring subsurface iron cations (5-fold coordinated).

We turn our attention to the experimental results for the highly reduced surface states of hematite, which were created by subjecting the stoichiometric Fe_2_O_3_(0001) to atomic hydrogen exposure followed by Ar^+^-sputtering and annealing cycles. Again, the changes in surface structure were monitored by measuring the CO vibrational frequency. Our IRRAS data allow to gain detailed insight into the surface structure of highly reduced α-Fe_2_O_3_(0001).

**Figure 4:**
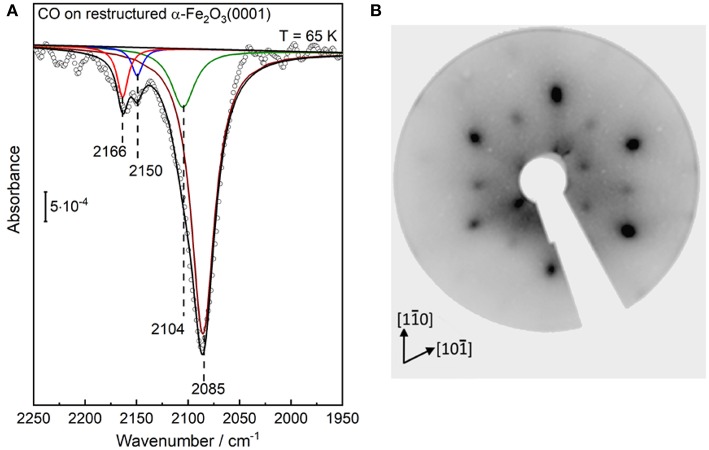
**(A)** IRRA spectrum (p-polarized) obtained after saturation adsorption of CO (1 L) at 65 K on a highly reduced α-Fe_2_O_3_(0001) surface created via atomic hydrogen treatment at RT followed by Ar^+^-sputtering and post-annealing at 950 K in 10^−6^ mbar oxygen. **(B)** LEED pattern of the corresponding highly reduced α-Fe_2_O_3_(0001) surfaces recorded at 90 eV.

After CO adsorption at 65 K, the p-polarized IRRAS data show a number of CO bands in the regions between 2,175–2,135 cm^−1^ and 2,125–2,025 cm^−1^ (Figure 4A), which are related respectively to CO species adsorbed on surface Fe^3+^ and Fe^2+^ sites, revealing substantial changes of the surface structure. To achieve a thorough analysis of the data, we subjected the IR spectra to a peak-fitting procedure starting with Lorentzian curves, which were centered at local maxima in the spectra. In the deconvoluted spectrum (Figure 4A), four distinct CO stretch bands are resolved at 2,166, 2,150, 2,104, and 2,085 cm^−1^ for highly reduced α-Fe_2_O_3_(0001), in full agreement with those obtained after CO adsorption on the reduced Fe_3_O_4_(111) surface [see section CO Adsorption on Fe_3_O_4_(111) and Fe_3_O_4_(001)].

Interestingly, Figure 4A shows that the intensity of the Fe^2+^-related CO bands is much stronger than that of the Fe^3+^-related CO vibrations at 2,166/2,150 cm^−1^. A higher population of CO species adsorbed to Fe^2+^ sites could account for this observation. However, the substantial intensity difference should be also related to a larger transition dipole moment for CO-Fe^2+^, resulting from the enhanced electron backdonation compared to CO/Fe^3+^, as confirmed by the large red-shift in frequency of CO species bound to Fe^2+^ cations. A similar phenomenon has been reported for CO adsorption on differently charged (Cu^+^ and Cu^2+^) copper ions, where the CO stretch intensity was calculated to be more than a factor of five higher for the CO-Cu^+^ than for the CO-Cu^2+^ complex (St. Petkov et al., 2012).

**Figure 5:**
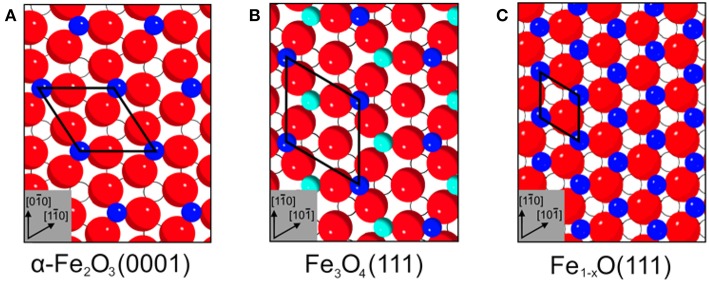
Top view surface structures of **(A)** α-Fe_2_O_3_(0001), **(B)** Fe_3_O_4_(111), and **(C)** Fe_1−x_O(111). The surface unit cells are **(A)** 5.04 Å, **(B)** 5.94 Å, and **(C)** 3.04 Å. Surface atoms are shown inked; subsurface atoms below the first oxide layer are depicted hollow. Red spheres correspond to oxide lattice ions; dark blue spheres and cyan spheres represent iron ions on octahedral and tetrahedral lattice sites, respectively.

Our IRRAS data strongly suggests that the highly reduced α-Fe_2_O_3_(0001) surface reconstructs. This conclusion is further supported by the corresponding LEED pattern (Figure 4B), which reveal that the α-Fe_2_O_3_(0001) surface undergoes a complete structural conversion from α-Fe_2_O_3_(0001) to Fe_3_O_4_(111) termination, in agreement with literature (Condon et al., 1994, 1998; Camillone et al., 2002). Furthermore, the outer hexagon spots become much broader and more intense, which could be attributed to the presence of additional, ordered Fe_1−x_O(111) domains [further detailed explanations are given in section CO Adsorption on Fe3O4(111) and Fe3O4(001)]. The surface structures of α-Fe_2_O_3_(0001), Fe_3_O_4_(111) and Fe_1−x_O(111) are illustrated in Figure 5. The LEED results indicate the formation of a Fe_3_O_4_(111)/Fe_1−x_O(111)-biphase structure on highly reduced α-Fe_2_O_3_(0001). The approach using non-destructive IR spectroscopy and CO as a probe molecule allows to provide more reliable information on the surface termination and chemical environments because CO only interacts with the outermost surface (Wang and Wöll, 2017; Chen et al., 2019). The CO vibrations at 2,166/2,104 cm^−1^ and 2,150 cm^−1^ could be attributed to octahedral Fe^3+^/Fe^2+^ sites as well as to tetrahedral Fe^3+^ sites within Fe_3_O_4_(111) domains, while the CO band the 2,085 cm^−1^ implies the coexistence of reduced Fe_1−x_O(111) islands terminated by octahedral Fe^2+^ cations. These assignments are corroborated by reference experiments where CO was adsorbed on Fe_3_O_4_(111) single-crystal surfaces, as discussed below in section CO adsorption on Fe_3_O_4_(111) and Fe_3_O_4_(001). Further evidence for this massive surface restructuring of the highly reduced α-Fe_2_O_3_(0001) is provided by photoelectron spectroscopy, where Fe^2+^ and Fe^3+^ species are clearly identified in both Fe 2p (Figure S5A) and valence band regions (Figure S5B) (Liu et al., 2012).

As mentioned in section Preview on Related Iron Oxide Crystal Structures, the highly reduced α-Fe_2_O_3_(0001) surface may also feature an ordered α-Fe_2_O_3_(0001)/Fe_1−x_O(111)-biphase reconstruction which is characterized by floret-like satellite spots, positioned adjacent to the peaks for the pristine (1 × 1)-α-Fe_2_O_3_(0001) surface (Condon et al., 1995, 1998). However, this surface reconstruction is not supported by our combined results from IRRAS and LEED (Figure 4). Our observation is in line with the STM studies on α-Fe_2_O_3_(0001) single crystal surfaces (Condon et al., 1998), where a Fe_3_O_4_(111)-termination was created by annealing the sample at ~1,000 K in 10^−6^ mbar O_2_. The structural transformation to a α-Fe_2_O_3_(111)/Fe_1−x_O(111)-biphase occurs only when increasing the temperature to about 1,100 K (Condon et al., 1998). In addition, more recent DFT calculations revealed that the α-Fe_2_O_3_(0001) surface can expose O-, Fe-, and ferryl-terminated regions, whose size varies depending on the oxygen chemical potential (Lewandowski et al., 2016). The long-range order was explained in terms of the dipole-dipole interaction between domains with different work functions.

### CO Adsorption on Fe_3_O_4_(111) and Fe_3_O_4_(001)

**Figure 6:**
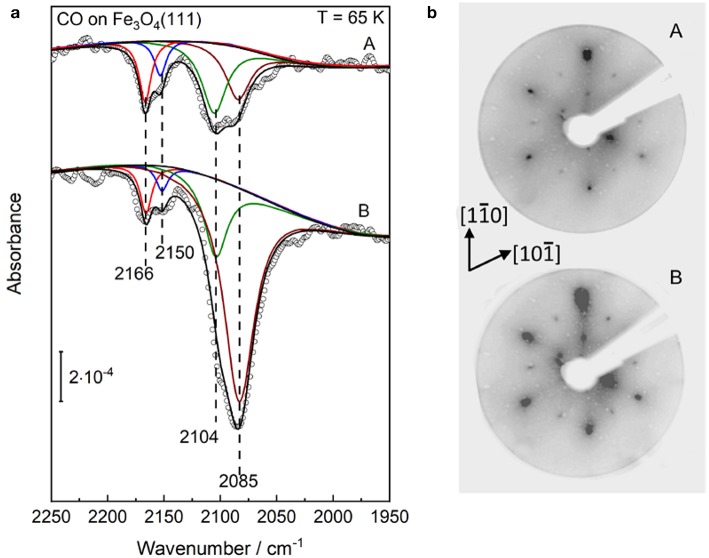
Structural evolution of well-defined Fe_3_O_4_(111) single-crystal surfaces after (A) slight and (B) heavy Ar^+^-sputtering treatment. Afterward such treatment the sample was annealed at 920 K in 10^−6^ mbar oxygen. **(a)** The p-polarized IRRAS data of CO adsorption on reduced Fe_3_O_4_(111) surfaces. The clean surfaces was saturated with a dose of 1 L CO at 65 K. **(b)** LEED patterns of the corresponding Fe_3_O_4_(111) surfaces recorded at 90 eV.

In order to aid the assignment of the CO bands and to gain a thorough understanding of the structural evolution of the α-Fe_2_O_3_(0001) surface during reduction, we have carried out additional reference measurements on Fe_3_O_4_(111) and reconstructed Fe_3_O_4_(001)-√2 × √2R45° (Figure S3) single crystal surfaces. Figure 6a shows p-polarized IRRAS data recorded after saturating the slightly (two sputter/annealing cycles) and strongly (five sputter/annealing cycles) reduced Fe_3_O_4_(111) single-crystal surfaces with CO at 65 K, which is much lower than the Verwey transition temperature of ~120 K (Verwey, 1939). The deconvoluted spectra display four CO stretch bands at 2,166, 2,150, 2,104, and 2,085 cm^−1^ (Figure 6a), in excellent agreement with the results found for the highly reduced surface of hematite α-Fe_2_O_3_(0001) (see Figure 4A).

As discussed in section Preview on Related Iron Oxide Crystal Structures, the surface structure of Fe_3_O_4_(111) is extremely sensitive to the preparation parameters (Kuhlenbeck et al., 2013; Parkinson, 2016). The experimental and theoretical studies revealed consistently that the regular Fe_3_O_4_(111) surface is Fe_tet1_-terminated over a close-packed oxygen layer, whereas this surface is stabilized by the Fe_oct2_-termination under oxygen-deficient conditions (Lennie et al., 1996; Ahdjoudj et al., 1999; Ritter and Weiss, 1999; Zhu et al., 2006; Grillo et al., 2008; Shimizu et al., 2010; Sala et al., 2012; Noh et al., 2015). The vibrational frequencies at 2,166/2,104 cm^−1^ and 2,150 cm^−1^ are assigned to CO species adsorbed to octahedral Fe^3+^/Fe^2+^ sites (B type) as well as to tetrahedral Fe^3+^ sites (A type), respectively, indicating a Fe_oct2_-terminated Fe_3_O_4_(111) structure (see Figure 5B).

The assignment of the CO bands is further assisted by additional IRRAS data obtained for CO adsorption on the highly reduced α-Fe_2_O_3_(0001) surface created by annealing at 950 K in a higher O_2_ pressure of 10^−4^ mbar (see Figure S6). In this condition, the Fe_3_O_4_(111) layer within the Fe_3_O_4_(111)/Fe_1−x_O biphase should be dominated by the Fe_tet1_-termination. Indeed, the corresponding IR data shows a single Fe^3+^-related vibrational band at 2,153 cm^−1^, which is attributed to CO molecules bound on surface Fe_tet1_ sites (see Figure S6).

Furthermore, we assign the low-lying IR band at 2,085 cm^−1^ to CO bound to Fe^2+^ surface sites from reduced Fe_1−x_O phases (see Figure 5C), as reported in the literature (Benziger and Larson, 1982). Again, the Fe_1−x_O(111) surface can expose either Fe or O layers according to the preparation conditions. Whereas, the STM investigations suggested the presence of O-terminated Fe_1−x_O(111) domains formed by oxidation of the Fe_3_O_4_(111) layer on α-Fe_2_O_3_(0001) (Tang et al., 2013), the DFT calculations predicted a Fe-termination in O-poor environments (Li et al., 2005). This assignment is supported by the observation that the 2,085 cm^−1^ band becomes more intense along with the Ar^+^-sputtering/annealing treatment of the Fe_3_O_4_(111) single-crystal surface (Figure 6a), indicating the growth of ordered Fe_1−x_O(111) domains, which coexist with the Fe_oct2_-terminated Fe_3_O_4_(111) structures on the reduced Fe_3_O_4_(111) surface. The stretch frequencies of all CO species adsorbed on different iron(III/II) oxide single-crystal surfaces studied in the course of this work are summarized in Table 1.

**Table 1:** CO stretch frequencies collected on regular and restructured hematite (0001) as well as on magnetite (111) and (001) single-crystal surfaces.

**Surface**	**Termination**	**CO stretch vibration (cm**^****−1****^**)**		
		**Fe^**3+**^(oct)**	**Fe^**3+**^ (tet)**	**Fe^**2+**^(oct)**
α-Fe_2_O_3_(0001)	Fe_oct_	2169	–	–
α-Fe_2_O_3−x_(0001)	Fe_oct_	2163	–	–
Fe_3_O_4_(111)	Fe_oct2_	2166	2150	2104
Fe_1−x_O(111)	Fe_oct_	–	–	2085
Fe_3_O_4_(001)	Fe_oct_O	2169		–

*The frequencies are shown for saturating CO exposures at 65 K*.

Our IR data provide direct spectroscopic evidence for the formation of a Fe_3_O_4_(111)/Fe_1−x_O(111) biphase structure on Fe_3_O_4_(111), in line with the results from STM and LEED (Condon et al., 1997; Shaikhutdinov et al., 1999; Paul et al., 2007). Here, the presence of a Fe_3_O_4_(111)/Fe_1−x_O(111) biphase is also supported by the LEED patterns shown in Figure 6B. In addition to the pristine Fe_3_O_4_(111) structure (Tarrach et al., 1993), the spots at the outer hexagon become much broader and more intense under reducing conditions, which is also seen for the highly reduced α-Fe_2_O_3_(0001) surface (see Figure 4B). This should originate from the presence of ordered Fe_1−x_O(111) domains. However, we did not see the floreted diffraction patterns as observed for a typical α-Fe_2_O_3_(0001)/Fe_1−x_O(111) biphase. This can be attributed to the rather small lattice mismatch (2%) between Fe_3_O_4_(111) and Fe_1−x_O(111), which makes an unambiguous observation of the floret-like satellite spots extremely difficult (Condon et al., 1997; Paul et al., 2007).

**Figure 7:**
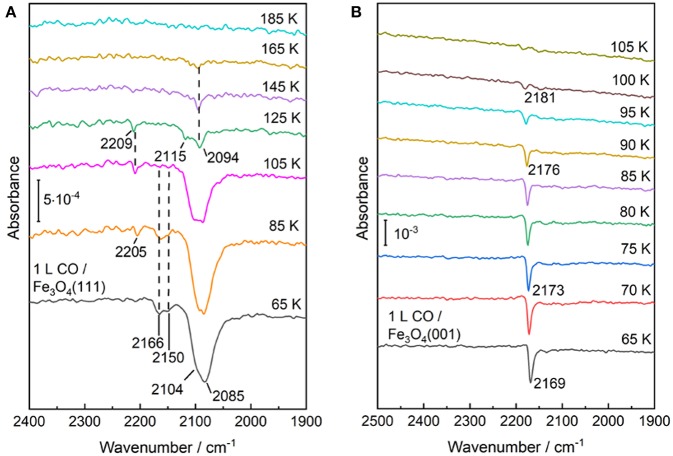
Temperature-dependent IRRA spectra starting from CO saturated surfaces of **(A)** Fe_3_O_4_(111) and **(B)** Fe_3_O_4_(001) at 65 K and subsequently heating to indicated temperatures. All the spectra were recorded at an incidence angle of 80° with p-polarized light at 65 K.

The temperature-dependent IRRAS data allows to gain deep insight into the interaction of CO molecule with various surface sites. As shown in Figure 7A, the Fe^3+^-related CO bands at 2,166 and 2,150 cm^−1^ disappear after annealing the sample to ~100 K, indicating a weak binding of CO to coordinatively unsaturated surface Fe^3+^ cations. The thermal stability and the frequency (blue-shifted compared to the gas phase value, see Table 1) are in good agreement with those observed on pristine α-Fe_2_O_3_(0001) (Figure 2B) and Fe_3_O_4_(001) surfaces (Figure 7B, see below), in which both are terminated by single Fe^3+^ species. In comparison, the Fe^2+^-related CO species at 2,104 and 2,085 cm^−1^ are thermodynamic more stable and desorb completely only upon annealing to temperatures above 165 K (Figure 7A). Again, the stronger binding of CO to Fe^2+^ is attributed to the enhanced electron back-donation to the CO 2π^*^ antibonding orbital, thus strengthening the CO-substrate interaction and weakening the internal C–O bond (redshift in frequency).

In addition, a new weak IR band at 2,205–2,209 cm^−1^ is detected after annealing to temperatures above 85 K. The origin of this band is not clear, but a similar CO species has been reported for ZnO and Au/ZnO systems and is assigned to CO coadsorbed with carbonate species that increase the Lewis acidity of adjacent Zn^2+^ cations (Wang et al., 2007; Noei et al., 2012). Carbonate could be produced via the oxidation of adsorbed CO with surface O atoms on Fe_3_O_4_(111) (Tinkle and Dumesic, 1987; Huang et al., 2006). In a recent study on Fe_3_O_4_(111) thin films grown on Pt(111) (Li et al., 2018), a CO vibration at 2,204 cm^−1^ was also observed. According to the DFT calculations, this band was ascribed to CO adsorbed at F${\text{e}}_{\text{oct}2}^{2+}$ ions located at step edges (defect sites). However, the Fe_3_O_4_(111) thin film, prepared under oxidation conditions, features a F${\text{e}}_{\text{tet}}^{3+}$-termination over close-packed oxygen layer, which is quite different from the present work focusing on the structural evolution of iron oxides under reducing conditions.

We now turn to the experimental results obtained on the reconstructed Fe_3_O_4_(001)-(√2 × √2)R45° surface. After adsorption of CO on the Fe_3_O_4_(001) surface at 65 K only one single sharp CO band at 2,169 cm^−1^ is observed (Figure 7B), which is typical for CO bound to Fe^3+^ sites. As discussed in section Preview on Related Iron Oxide Crystal Structures, the (√2 × √2)R45°-reconstructed Fe_3_O_4_(001) surface has been studied extensively by numerous groups (Wiesendanger et al., 1992; Tarrach et al., 1993; Chambers and Joyce, 1999; Rustad et al., 1999; Stanka et al., 2000; Mariotto et al., 2002; Parkinson et al., 2011; Bartelt et al., 2013; Bliem et al., 2014). Recently, Parkinson et al. (Bliem et al., 2014) presented a thorough study combining STM, LEED *IV* analysis and theory, in which a subsurface cation vacancy (SCV) model was developed. According to this model, the reconstructed surface is Fe_oct_-O terminated, which is accompanied by a rearrangement of the cations in the subsurface layers (Bliem et al., 2014).

Our IRRAS results are fully consistent with the SCV structure in that the single IR band at 2,169 cm^−1^ is characteristic for CO bound to surface F${\text{e}}_{\text{oct}}^{3+}$ sites. The assignment to Fe^3+^ is further supported by a temperature-programmed desorption measurement, where the IRRAS data reveal a complete desorption of CO species after heating to 105 K, in excellent agreement with the TPD results (Hulva et al., 2018). This finding demonstrates again a weak interaction between CO and surface Fe^3+^ cations as observed for CO adsorption on the Fe^3+^ sites of α-Fe_2_O_3_(0001) and Fe_3_O_4_(001) surfaces. In addition, the thermal desorption of CO leads to a blue shift in frequency from 2,169 to 2,181 cm^−1^ (Figure 7B), which is attributed to the lateral adsorbate-adsorbate interactions [for a detailed discussion see section CO Adsorption on the Pristine α-Fe_2_O_3_(0001) Surface].

Overall, the IRRAS results, acquired for CO adsorption on the macroscopic Fe_3_O_4_(111) and Fe_3_O_4_(001) surfaces, provide a solid basis for a reliable assignment of the CO bands observed for the restructured α-Fe_2_O_3_(0001) surface under different reduction conditions (Table 1). Importantly, the comprehensive IR data, together with XPS and LEED analysis, allow for identifying the Fe_3_O_4_(111)/Fe_1−x_O(111) biphase structure on highly reduced α-Fe_2_O_3_(0001) surfaces.

## Conclusions

In summary, the structural evolution of differently treated iron(III/II) oxide single-crystal surfaces was monitored by polarization-resolved IRRAS using CO as a probe molecule. We presented a comprehensive investigation on stoichiometric and reduced α-Fe_2_O_3_(0001) surfaces as well as on Fe_3_O_4_(001) and Fe_3_O_4_(111) surfaces. The polarization-dependent IRRAS results demonstrate that the CO adsorption is well suited to monitor the atomic structure evolution of iron oxide surfaces under different conditions.

The pristine stoichiometric α-Fe_2_O_3_(0001) surface is terminated in a single-Fe^3+^ configuration as characterized by the single sharp IR band at 2,169 cm^−1^ (CO on 3-fold coordinated Fe^3+^). Annealing the hematite (0001) surface to elevated temperatures leads to the formation of surface oxygen vacancies. The reduced α-Fe_2_O_3_(0001) surface shows a typical vibrational band at 2,163 cm^−1^ (CO on Fe^3+^ at O vacancy). A further reduction of α-Fe_2_O_3_(0001) induced by atomic hydrogen treatment followed by Ar^+^-sputtering and annealing cycles under oxygen poor conditions causes a massive surface restructuring yielding a Fe_3_O_4_(111) overlayer, which coexists with Fe_1−x_O(111) domains. The reconstructed Fe_3_O_4_(111) surface is Fe_oct2_-terminated and shows typical CO bands at 2,166/2,150 and 2,104 cm^−1^. They are assigned to Fe^3+^(oct)/Fe^3+^(tet) and Fe^2+^(oct) sites, respectively. The Fe_1−x_O(111) domains within the Fe_3_O_4_(111)/Fe_1−x_O(111) biphase are characterized by the intense band at 2,085 cm^−1^ originating from CO adsorbed on Fe^2+^(oct) sites. The assignment is further demonstrated by reference IRRAS data acquired for CO adsorption on Fe_3_O_4_(111) single-crystal surfaces. The surface of Fe_3_O_4_(001) is terminated with F${\text{e}}_{\text{oct}}^{3+}$-O in a SCV reconstruction as determined by the single intense IR band of CO at 2,169 cm^−1^.

## Data Availability

All datasets generated for this study are included in the manuscript and/or the Supplementary Files.

## Author Contributions

YW and CW conceived the idea of the experiment. LS and CY carried out the experiments, with additional help by AN and SH. Data analysis was carried out by LS, CY, and YW. The manuscript was written by LS, YW, and CW.

### Conflict of Interest Statement

The authors declare that the research was conducted in the absence of any commercial or financial relationships that could be construed as a potential conflict of interest.

## Supplementary Material

Additional XPS, LEED, and polarization-resolved IRRAS analysis.
