# Supplementary material for: Structural Evolution of α-Fe2O3(0001) Surfaces Under Reduction Conditions Monitored by Infrared Spectroscopy
Source: Front Chem. 2019 Jun 25;7:451. doi: 10.3389/fchem.2019.00451 (PMC6603135; doi:10.3389/fchem.2019.00451)
Supplement: Supplementary file 1 [file Data_Sheet_1.pdf]

SUPPORTING INFORMATION

Structural Evolution of α-Fe_2O_3(0001) Surfaces under Reduction Conditions Monitored by Infrared Spectroscopy

Ludger Schöttner, Alexei Nefedov, Chengwu Yang, Stefan Heissler, Yuemin Wang, Christof Wöll**

Institute of Functional Interfaces (IFG), Karlsruhe Institute of Technology (KIT), 76344 Eggenstein-Leopoldshafen, Germany

*E-mails: yuemin.wang@kit.edu; christof.woell@kit.edu

1. Near edge X-ray absorption fine spectra (NEXAFS)

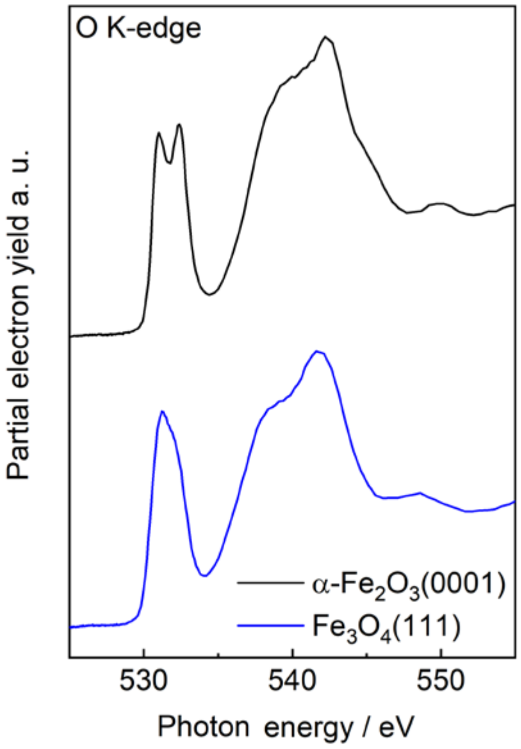

Figure S1: O K-edge NEXAFS spectra recorded on clean α-Fe_2O_3(0001) and Fe_3O_4(111) surfaces.

For α-Fe_2O_3(0001), there are two components on the pre-peak around 532 eV of approximately equal intensity, clearly separated by 1.3 eV, whereas for Fe_3O_4(111), the second feature appears only as a shoulder at +0.7 eV in agreement with literature (Figure S1).[1] The pre-peak is commonly associated with the excitations of O 1s electrons into O 2p states hybridized with Fe 3d orbitals. This orbital hybridization causes a symmetry related ligand field splitting in e_g and t_{2g} energy levels on d-electrons.[2, 3, 4] In such scenario, one might expect for ferric oxide, which contains Fe^{3+} cations only, an intensity ratio of 3:2 on Fe d^5 electron configurations. However, the X-ray absorption spectra on α-Fe_2O_3(0001) do not agree in this point, which is oftentimes mentioned as an intensity problem. The common interpretation of this problem was that for e_g electrons the O 2p – Fe 3d hybridization is stronger and this causes the higher intensity of the corresponding feature including exchange splitting effects.[2, 5]

2. Supplementary low-energy electron diffraction (LEED) data

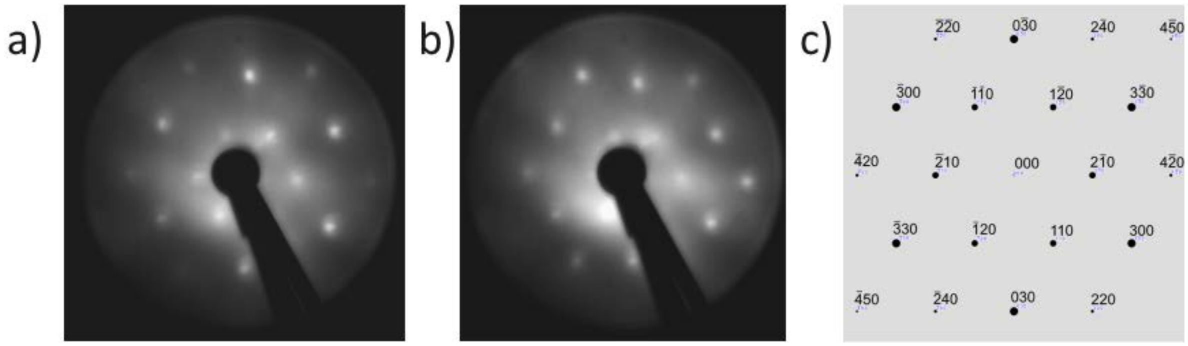

Figure S2: LEED patterns recorded on α-Fe_2O_3(0001) at a) 90 eV and b) 120 eV. c) Simulation of an electron-weighted LEED pattern for α-Fe_2O_3(0001) surfaces.

The LEED pattern were obtained on the pristine surface of α-Fe_2O_3(0001) and agree perfectly with literature data.[6] The foregrounded beam intensity at the (1×1)-peaks, measurable on energies around 90 eV for incident electrons, is a characteristic feature for the hematite (0001) unit cell symmetry and indicates a high surface activity and cleanliness. This suggests the electron-weighted LEED pattern from a computed α-Fe_2O_3(0001) structure due to the resemblance with the experimental pattern (Figure S2a-c).

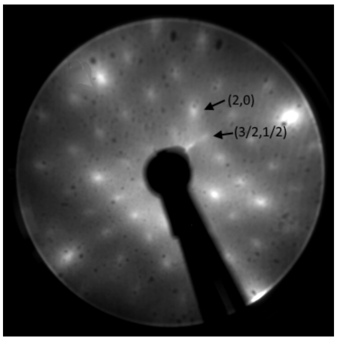

Figure S3: LEED pattern recorded on a Fe_3O_4(001) surface at 90 eV.

The LEED pattern for the reconstructed (√2×√2)R45° Fe_2O_3(001) surface (Figure S3) comprises a specific "fingerprint", which let one distinguish between a SCV structure and a

“Fe-dimer” structure.[7] In case the half-order spots (e.g 3/2,1/2) occur in a similar intensity to the integer spots (e.g. 2,0), the SCV structure is obtained. In the “Fe-dimer” structure the (√2×√2)R45°-related spots are significantly weakened. Here, we assigned the LEED pattern to a SCV structure.

3. Calculation and influence of lateral interactions on the vibrational spectra of CO/α-Fe$_2$O$_3$(0001) systems

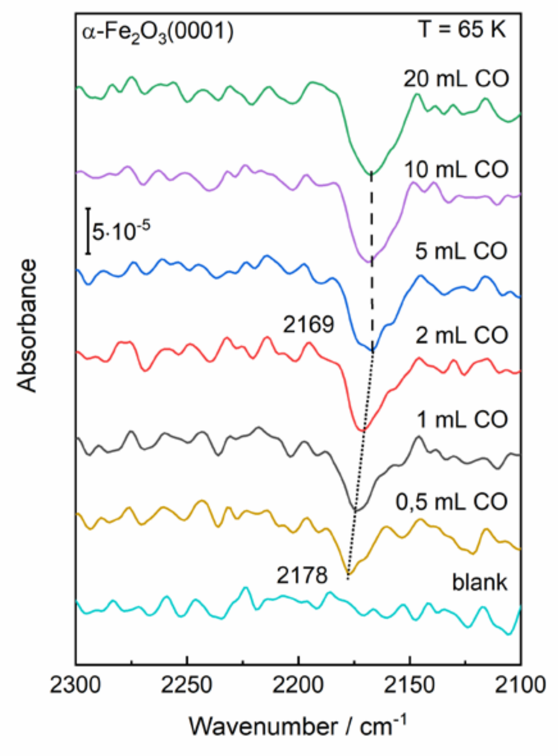

Figure S4: IRRA spectra obtained after exposing pristine α-Fe$_2$O$_3$(0001) to different doses of CO at 65 K. The spectra were recorded at an incidence angle of 80° with p-polarized light.

In the past, attempts have been made to mathematically define coverage-dependent wavenumber shifts on CO adsorbates. Coupling contributions $\Delta\omega_{dyn} = \omega - \omega_0$ between adsorbed CO molecules can be approximated using the modified Hammaker equation (dynamic shift).[8, 9, 10, 11]

$$\omega = \omega_0 \left(1 + \frac{\alpha_v \theta \sum_0}{1 + \alpha_e \theta \sum_0}\right)^{1/2}$$

The calculation of the coupling contribution is now carried out using the example of CO on α-Fe_2O_3(0001). The wavenumber ω_0 of an isolated CO oscillation ($\theta \rightarrow 0$) was experimentally determined with 2178 cm^{-1} (Figure S4). Here the polarizability coefficients $\alpha_{\|}$ = 2,6 $Å^3$, which applies to an E-field vector in IR radiation aligned parallel to the CO molecule axis, was used to calculate α_e = 2,573 $Å^3$ with help of $\alpha_{\|} = \alpha_v + \alpha_e$ and α_v = 0,027 $Å^3$.[11, 12] The dipole sum $\sum_0 = T+V$ is composed of the direct dipole sum $T = Cn^{3/2}$ and the image dipole V. An image dipole was not considered on the dielectric substrate. The expression $n = 2/\sqrt{3}a^2$ for describing the atomic density of the adsorption sites was determined by the trigonal symmetry of hematite using the lattice constant a = 5,04 Å.[10, 13] C is a constant with the size 8,89.[10] The direct dipole sum T was calculated to be 0,086 $Å^{-3}$. The coupling contribution to the wavenumber shift $\Delta\omega_{dyn}$ is thus 2.0 cm^{-1} in the case of a completely covering surface, i.e. using θ = 1 ML CO on α-Fe_2O_3(0001). The calculated interval to the coupling contribution is comparatively small in contrast to the total measured frequency difference. As a result, the observed frequency shift is mostly caused by a direct adsorbate-substrate interaction (static shift). Our calculated value is in good agreement with the previous reported value of 1.5 cm^{-1}, which was obtained on CO covered α-Fe_2O_3 powder samples.[11]

4. X-ray photoemission spectra (XPS)

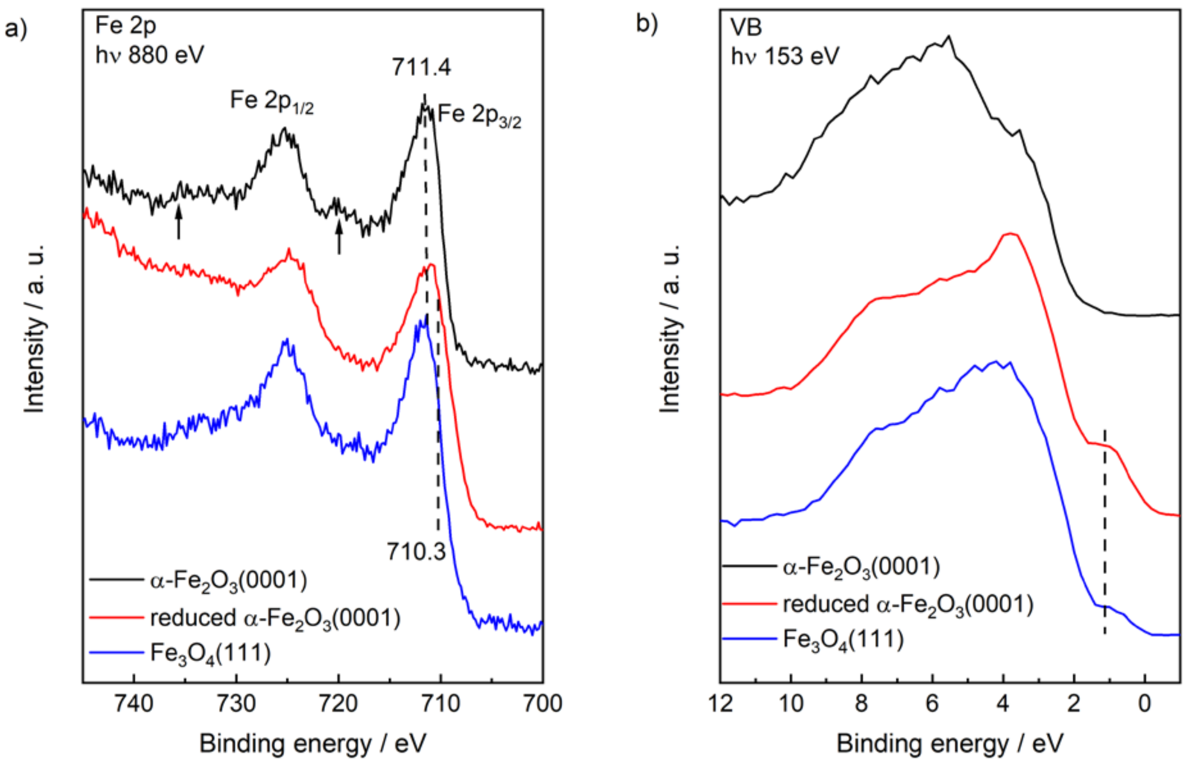

Figure S5: Comparitive presentation of photoelectron spectra for α-Fe_2O_3(0001), strongly reduced α-Fe_2O_3(0001) and sputter/annealed Fe_3O_4(111) surfaces in a) Fe 2p and b) valence band region.

The indicated BEs of Fe 2p3/2 lines of Fe^{3+} and Fe^{2+} states at 711.4 and 710.3 eV are around the reported reference energies of 711.0 and 710.0 eV with satellites (in Figure S5a marked with arrows) respectively.[14,15,16] We used a binding energy of 530.0 eV to determine Fe 2p core level binding energies. The prepared α-Fe_2O_3(0001) surface only consists Fe^{3+} cationic states and was identified by a centered line at 711.4 eV on Fe $2p_{3/2}$ core level including a satellite. The emerge of Fe^{2+} is traced by additional Fe 2p lines, which can be correspondingly observed by a strong weakening of the satellite peaks.[17] The electronic structure of a strongly reduced hematite sample is in overall agreement with the sputter/annealed Fe_3O_4(111) surface. Fe^{2+} species on reduced hematite were further tracked in the valence band region (Figure S5b). Here,

the VB spectrum shows a peak at 1 eV, which originates from Fe $3d^6$ final states, associated with Fe^{2+} ions.[18] However a clear separation of certain Fe 3d states remains complex and is mainly assumed to result from a broad photoemission contribution of O 2p states between 2-8 eV.[19,20]

5. Supplementary IRRAS data

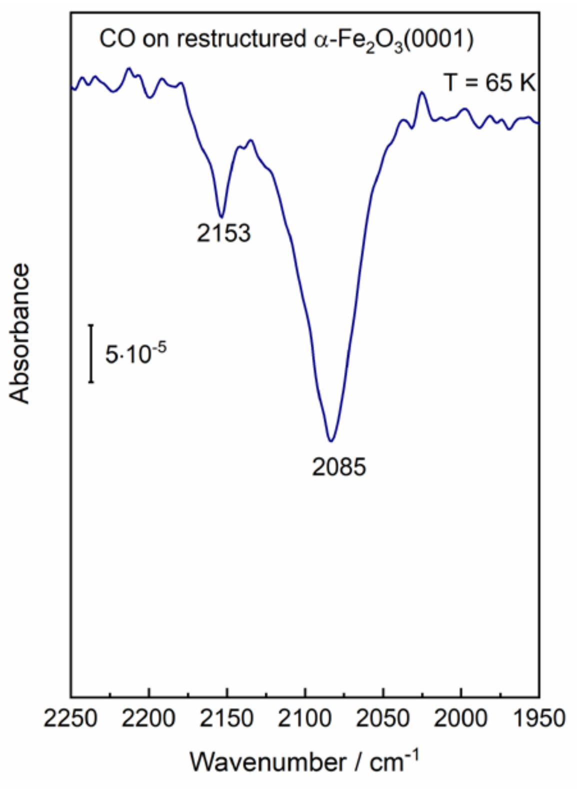

Figure S6: IRRA spectrum (p-polarized) obtained after saturation adsorption of CO (1 L) at 65 K on a highly reduced α-Fe_2O_3(0001) surface created via atomic hydrogen treatment at RT followed by Ar^+-sputtering and post-annealing at 950 K in 10^{-4} mbar oxygen.

According to STM the boundaries of several surface terminations of Fe_3O_4(111) are quite close together and tend to manifest in equilibration with the oxygen environment, so it should not wonder that the surface structure of Fe_3O_4(111) can be transformed from Fe_{tet1} termination to Fe_{oct2}-termination when the partial pressure of oxygen is lowered during crystal preparation.[21] This is a crucial point, which needs to be considered carefully when discussing the surface stability of Fe_3O_4(111). According to some DFT studies the Fe_{oct2}-terminated Fe_3O_4(111) surface is only preferable for decreased values of the oxygen-chemical potential [22] or only little

more stable than Fe_{tet1}-termination over a wide range of oxygen pressures.[23] When the sputter-reduced surface of α-Fe_2O_3(0001) is prepared under more oxidizing conditions (10^{-4} mbar O_2, 950 K) as given in the main script, the surface structure is dominated by a Fe_{tet1}-terminated Fe_3O_4(111) structure within the Fe_3O_4(111)/$Fe_{1-x}O$ biphase as indicated by a single Fe^{3+}-related vibrational band at 2150 cm^{-1}, which correspond to linearly adsorbed CO molecules on tetrahedral Fe^{3+} ions (Figure S6). After this preparation routine, Fe_{oct2} species only occur in minor fractions on Fe_3O_4(111) surface domains as confirmed by the absence of vibrational bands at 2166 cm^{-1} and 2106 cm^{-1}, which would be associable to octahedral Fe^{3+} and Fe^{2+} surface ions from Fe_3O_4(111), respectively. The other sharp vibrational band at 2085 cm^{-1} correspond to octahedral Fe^{2+} ions from iron terminated $Fe_{1-x}O$(111) surface. This result is in excellent agreement with the experimental findings and propositions of the Osgood group[24,25] as well as of the Thornton group.[26]

[1] Z. Y. Wu, S. Gota, F. Jollet, M. Pollak, M. Gautier-Soyer, C. R. Natoli, *Phys. Rev. B* **1997**, 55, 4, 2570-2577.
[2] T.-J. Park, S. Sambasivan, D. A. Fischer, W.-S. Yoon, J. A. Misewich, S. S. Wong, *J. Phys. Chem. C* **2008**, 112, 10359-10369.
[3] F. M. F. de Groot, M. Grioni, J. C. Fuggle, *Phys. Rev. B* **1989**, 40, 8, 5715-5723.
[4] J. H. Paterson, O. L. Krivanek, *Ultramicroscopy* **1990**, 32, 319-325.
[5] I. Leonov, A. N. Yaresko, V. N. Antonov, V. I. Anisimov, *Phys. Rev. B* **2006**, 74, 165117, 1-14.
[6] W. Weiss, W. Ranke, Prog. Surf. Sci. 2002, 70, 1-151.
[7] G. Parkinson, Surf. Sci. Rep. 2016, 71, 272-365.
[8] M. Buchholz, X. Yu, C. Yang, S. Heißler, A. Nefedov, Y. Wang, C. Wöll, *Surf. Sci.* **2016**, 662, 247-252,
[9] P. Hollins, J. Pritchard, *Chem. Phys. Lett.* **1980**, 75, 2, 378-382.
[10] G. D. Mahan, A. A. Lucas, *J. Chem. Phys.* **1978**, 68, 1344-1348.
[11] A. Zecchina, D. Scaranao, A. Reller, *J. Chem. Soc.*, *Faraday Trans. 1*, **1988**, 84, 7, 2327-2333.
[12] M. Scheffler, *Surf. Sci.* **1979**, 81, 562-570.
[13] R. L. Blake, R. E. Hessevick, *Am. Mineral.* **1966**, 51, 123-129.
[14] T. Yamashita, P. Hayes, *Appl. Surf. Sci.* **2008**, 254, 2441-2449.
[15] W. Weiß, *Surf. Sci.* **1997**, 377-379, 943-947
[16] R. L. Kurtz, V. E. Henrich, *Surf. Sci.* **1983**, 129, 345-354.
[17] Th. Schedel-Niedrig, W. Weiss, R. Schlögl, *Phys. Rev. B* **1995**, 52, 24, 17449-17460.
[18] S. Liu, S. Wang, J. Guo, Q. Guo, *RSC Advances* **2012**, 2, 9938-9943.
[19] R. J. Lad, V. E. Henrich, *Phys. Rev. B* **1989**, 39, 18, 13478-13485.
[20] A. Fujimori, M. Saeki, N. Kimizuka, M. Taniguchi, S. Suga, *Phys. Rev. B* **1986**, 34, 10, 7318-7328.
[21] T. K. Shimizu, J. Jung, H. S. Kato, Y. Kim, M. Kawai, Phys. Rev. B, 2010, 81, 235429.
[22] J. Noh, O. Osman, S. G. Aziz, P. Winget, J.-L. Bredás, *Chem. Mater.* **2015**, 27, 5856-5867.
[23] L. Zhu, K. L. Yao, Z. L. Liu, *Phys. Rev. B* 2006, 74, 035409.
[24] N. Camillone III, K. Adib, J. P. Fitts, K. T. Rim, G. W. Flynn, S. A. Joyce, R. M. Osgood, *Surf. Sci.* 2002, 511, 267-282.
[25] K. T. Rim, J. P. Fitts, T. Müller, K. Adib, N. Camillone III, R. M. Osgood, S. A. Joyce, G. W. Flynn, Surf. Sci. 2003, 541, 59-75.
[26] N. G. Condon, F. M. Leibsle, A. R. Lennie, P. W. Murray, T. M. Parker, D. J. Vaughan, G. Thornton, Surf. Sci. 1998, 397, 278-287.
